# Supplementary material for: Timberline structure and woody taxa regeneration towards treeline along latitudinal gradients in Khangchendzonga National Park, Eastern Himalaya
Source: PLoS One. 2018 Nov 28;13(11):e0207762. doi: 10.1371/journal.pone.0207762 (PMC6261585; doi:10.1371/journal.pone.0207762)
Supplement: S1 Table — (DOCX) [file pone.0207762.s001.docx]

| Sn | Forest | Elevation range  (m asl) | Type | Sub-Type |
| --- | --- | --- | --- | --- |
| 1 | East Himalayan Sub-tropical wet hill Forest | 1000-1800 | 8B/C_1_ |  |
| 2 | Lauracxeous forest | 800-2100 |  | 11B/C_1_ a |
| 3 | Buk-oak forest | 2100-2440 |  | 11 B/C_1_ b |
| 4 | High-level Oak Forest | 2440-2750 |  | 11B/C_1_ c |
| 5 | East Himalayan mixed conifer forest | 2300-3000 |  | 12/C_3_ a |
| 6 | East Himalayan dry temperate conifer forest | 2800-4500 | 13/C_6_ |  |
| 7 | East Himalayan sub-alpine birch/fir forest | above 3000 | 14/C_2_ |  |
| 8 | Birch-Rhododendron scrub Forest | above 3500 | 15/C_1_ |  |
| 9 | Decidious alpine scrub | above 3500 | 15/C_2_ |  |
| 10 | Alpine Pasture | above 4000 | 15/C_3_ |  |

**S1 Table.** Forest types/sub-types of Khangchendzonga National Park according to the revised classification of Champion and Seth [43]
