# Supplementary material for: Timberline structure and woody taxa regeneration towards treeline along latitudinal gradients in Khangchendzonga National Park, Eastern Himalaya
Source: PLoS One. 2018 Nov 28;13(11):e0207762. doi: 10.1371/journal.pone.0207762 (PMC6261585; doi:10.1371/journal.pone.0207762)
Supplement: S2 Table — (DOCX) [file pone.0207762.s002.docx]

**S2 Table.** Composition of woody taxa across the timberline of Khangchendzonga National Park

| **SN** | **Botanical name** | **Habit** | **Family** | **English name** | **Local name** |
| --- | --- | --- | --- | --- | --- |
| 1 | *Abies densa* Griff*.* | Tree | Pinaceae | The Indian Silver Fir | Gobre Salla |
| 2 | *Prunus rufa* Wall. | Tree | Rosaceae | Small Himalayan cherry | Arupate |
| 3 | *Sorbus microphylla* Decne*.* | Tree | Rosaceae |  | Sanupasi |
| 4 | *Rhododenderon arboretum* Sm*.* | Tree | Ericaceae | Scarlet arbosescent Rhododendron | Lali gurans |
| 5 | *Rhododenderon hodgsonii* Hook.f. | Tree | Ericaceae | Hodgson’s Rhododendron | Gulabi Korliga |
| 6 | *Rhododendron lanatum* Hook.f. | Tree | Ericaceae | Wooly rhododendron | Bhutle gurans |
| 7 | *Rhododendron wightii* Hook.f. | Tree | Ericaceae | Dr. Wight’s Rhododendron | Dr. Wight ko gurnas |
| 8 | *Rhododenderon thomsonii* Hook.f. | Tree | Ericaceae | Dr. Thomson’s Rhododendron | Dr. Thomson ko gurnas |
| 9 | *Pieris villosa* Hook.f. | Tree | Ericaceae |  | Angari |
| 10 | *Virburnum cordifolium* Wall ex. DC | Tree | Caprifoliaceae |  | Asare |
| 11 | *Rhododendron fulgens* Hook.f. | Tree | Ericaceae | Brilliant Rhododendron | Chimal |
| 12 | *Juniperus recurva* Buch. Ham ex. D. Don | Shrub | Cupressaceae |  | Dhupi |
| 13 | *Rhododendron campanulatum* D. Don | Shrub | Ericaceae | Bell flowered Rhododendron | Nilo chimal |
| 14 | *Rhododendron lepidotum* Wall. ex G. Don | Shrub | Ericaceae | Scaly Rhododendron | Bhale sunpate |
| 15 | *Rhododendron setosum* D. Don | Shrub | Ericaceae | Bristly Rhododendron | Tsallu gurans |
| 16 | *Rhododendron anthopogon* D. Don | Shrub | Ericaceae | Bearded Rhododendron | Dhupi gurans |
| 17 | *Ribes glaciale* Wall. | Shrub | Grossulariaceae |  | Robhay |
| 18 | *Rosa sericia* Wall. | Shrub | Rosaceae | Himalayan rose |  |
| 19 | *Gaultheria trichophylla* Royle | Shrub | Ericaceae |  |  |
| 20 | *Gaultheria pyroloides* Hook.f. | Shrub | Ericaceae | Japanese winter green |  |
