# Supplementary material for: Timberline structure and woody taxa regeneration towards treeline along latitudinal gradients in Khangchendzonga National Park, Eastern Himalaya
Source: PLoS One. 2018 Nov 28;13(11):e0207762. doi: 10.1371/journal.pone.0207762 (PMC6261585; doi:10.1371/journal.pone.0207762)
Supplement: S3 Table — Values in parenthesis indicate importance value index; values followed by the same letters within a column are not significantly (p<0.05) differ to each other; C: Simpson’s index of dominance; H: Shannon-Weiner diversity Index; I: Margalef’s Index of species richness; E: Shannon Index of species evenness. (DOCX) [file pone.0207762.s003.docx]

**S3 Table.** Ecological attributes of timberline vegetation of Khangchendzonga National Park

| Sites | Vegetation community | | Tree | | | | Shrubs | | | |
| --- | --- | --- | --- | --- | --- | --- | --- | --- | --- | --- |
|  | Tree | Shrubs | C | H | I | E | C | H | I | E |
| S1 | *Abies densa* (172.68) | *Rosa sericea* (172.36) | 0.42^ab^ | 1.01^b^ | 3.02^de^ | 0.87^ab^ | 0.50^ab^ | 0.75^ab^ | 2.45^ab^ | 0.87^a^ |
| S2 | *Abies densa* (119.23*), Rhododendron lanatum*(102.17) | *Rosa sericea* (64.78); *Ribes glaciale*(52.93) | 0.38^b^ | 1.08^b^ | 3.29d | 0.89^ab^ | 0.43^ab^ | 0.96^ab^ | 2.74^ab^ | 0.93^a^ |
| S3 | *Abies densa* (112.42), *Sorbus microphylla* (102.07) | *Rosa sericea* (182.85) | 0.37^b^ | 1.10^b^ | 3.58^cd^ | 0.84^ab^ | 0.55^ab^ | 0.72^ab^ | 2.41^ab^ | 0.77^a^ |
| S4 | *Abies densa* (120.81), *Rhododendron lanatum* (81.27) | *Rosa sericea* (227.65) | 0.37^b^ | 1.15^b^ | 4.37^bc^ | 0.77^cd^ | 0.68^a^ | 0.51^b^ | 1.86^b^ | 0.53^a^ |
| S5 | *Rhododendron lanatum* (89.53), *Sorbus microphylla* (87.01), *Abies densa* (68.29) | *Rosa sericea* (136.08) | 0.37^b^ | 1.21^b^ | 5.04^ab^ | 0.74^d^ | 0.41^ab^ | 0.97^ab^ | 2.68^ab^ | 0.93^a^ |
| S6 | *Abies densa* (111.12) | *Rhododendron campanulatum*(95.78) | 0.25^c^ | 1.47^a^ | 4.80^b^ | 0.92^a^ | 0.42^ab^ | 1.00^ab^ | 3.26^ab^ | 0.81^a^ |
| S7 | *Sorbus microphylla* (73.51), *Rhododendron wightii* (64.30), *R. lanatum*(61.36), *R. thomsonii*(55.68), *Abies densa*(45.14) | *Rosa sericea* (118.06) | 0.39^b^ | 1.09^b^ | 3.62^cd^ | 0.82^bc^ | 0.41^ab^ | 0.99^ab^ | 2.74^ab^ | 0.51^a^ |
| S8 | *Sorbus microphylla* (79.52)*, R. hodgsonii(*70.43*) Abies densa* (50.28) | *Ribes glaciale* (151.57); *Rosa sericea* (114.99) | 0.27^c^ | 1.51^a^ | 5.79^a^ | 0.89^ab^ | 0.41^ab^ | 0.99^ab^ | 2.76^ab^ | 0.49^a^ |
| S9 | *Rhododendron wightii* (155.71) | *Rosa sericea* (58.37), *Rhododendron campanulatum* (56.84) | 0.49^a^ | 0.77^c^ | 2.16^e^ | 0.91^a^ | 0.32^b^ | 1.29^a^ | 4.36^a^ | 0.39^a^ |

Values in parenthesis indicating importance value index; values followed by same letters within a column are not significantly *(p<0.05)* differ each other C: Simpson’s index of dominance; H: Shannon-Weiner diversity Index; I: Margalef’s Index of species richness; E: Shannon Index of species evenness
