# Supplementary material for: Timberline structure and woody taxa regeneration towards treeline along latitudinal gradients in Khangchendzonga National Park, Eastern Himalaya
Source: PLoS One. 2018 Nov 28;13(11):e0207762. doi: 10.1371/journal.pone.0207762 (PMC6261585; doi:10.1371/journal.pone.0207762)
Supplement: S4 Table — Total tree density values within a column and average tree density value within a row followed by the same letters are not significantly (p<0.05) different from each other. (DOCX) [file pone.0207762.s004.docx]

**S4 Table.** Tree density (individual ha^-1^) across different Dzongri timberline sites of Khangchendzonga National Park

|  | *Abies*  *densa* | *Sorbus*  *microphylla* | *Rhododendron*  *lanatum* | *Rhododendron*  *hodgsonii* | *Rhododendron*  *wightii* | *Rhododendron*  *fulgens* | *Rhododendron*  *arboreum* | *Rhododendron*  *thomsonii* | *Prunus*  *rufa* | *Piris*  *villosa* | Total |
| --- | --- | --- | --- | --- | --- | --- | --- | --- | --- | --- | --- |
| **Site 1** | 87.56±27.56 | 91.33±27.72 | 81.67±40.86 | 0.00±0.00 | 0.00±0.00 | 0.00±0.00 | 0.00±0.00 | 0.00±0.00 | 26.67±17.64 | 0.00±0.00 | 287.22±89.37^bc^ |
| **Site 2** | 60.0±20.0 | 125.0±35.0 | 126.0±14.0 | 0.00±0.00 | 50.00±50.0 | 0.00±0.00 | 0.00±0.00 | 0.00±0.00 | 15.00±15.00 | 0.00±0.00 | 376.0±104.0^bc^ |
| **Site 3** | 55.33±11.84 | 166.44±49.41 | 70.22±15.71 | 0.00±0.00 | 0.00±0.00 | 0.00±0.00 | 0.00±0.00 | 0.00±0.00 | 15.00±15.00 | 0.00±0.00 | 307.0±71.63^bc^ |
| **Site 4** | 131.11±34.50 | 110.67±100.83 | 125.33±23.59 | 0.00±0.00 | 56.67±12.02 | 33.33±33.33 | 0.00±0.00 | 0.00±0.00 | 40.00±40.00 | 0.00±0.00 | 497.11±93.54^ab^ |
| **Site 5** | 102.0±4.16 | 207.33±86.17 | 186.33±16.05 | 0.00±0.00 | 86.67±16.91 | 20.00±11.55 | 0.00±0.00 | 0.00±0.00 | 126.67±107.29 | 6.67±06.67 | 735.67±161.67^a^ |
| **Site 6** | 80.0±36.0 | 112.0±28.0 | 100.00±40.0 | 72.00±28.0 | 0.00±0.00 | 0.00±0.00 | 0.00±0.00 | 0.00±0.00 | 50.00±30.00 | 0.00±0.00 | 414.0±106.0^bc^ |
| **Site 7** | 33.33±24.04 | 26.67±16.22 | 65.00±05.0 | 0.00±0.00 | 52.0±26.03 | 0.00±0.00 | 0.00±0.00 | 45.0±22.91 | 0.00±0.00 | 0.00±0.00 | 222.0±35.02^bc^ |
| **Site 8** | 22.67±2.67 | 70.11±21.71 | 62.22±21.89 | 126.0±23.86 | 66.67±17.64 | 0.00±0.00 | 36.67±20.28 | 0.00±0.00 | 20.00±11.55 | 6.67±06.67 | 411.0±32.79^bc^ |
| **Site 9** | 0.00±0.00 | 28.89±17.36 | 16.67±16.67 | 0.00±0.00 | 49.33±10.97 | 0.00±0.00 | 0.00±0.00 | 0.00±0.00 | 25.00±12.58 | 0.00±0.00 | 119.89±32.04^c^ |
| **Average** | 63.56±13.74^bc^ | 104.27±19.74^a^ | 92.60±16.29^ab^ | 22.0±15.23^e^ | 40.15±10.74^cd^ | 5.93±4.07^de^ | 4.07±4.07^de^ | 05.0±05.0^de^ | 35.37±12.40^cde^ | 1.48±0.98^e^ | 373.69 |

Total tree density values with in a column and average tree density value within a row followed by same letters are not significantly (*p<0.05*) different from each other
