# Supplementary material for: Timberline structure and woody taxa regeneration towards treeline along latitudinal gradients in Khangchendzonga National Park, Eastern Himalaya
Source: PLoS One. 2018 Nov 28;13(11):e0207762. doi: 10.1371/journal.pone.0207762 (PMC6261585; doi:10.1371/journal.pone.0207762)
Supplement: S5 Table — Total shrub density values with in a column and average shrub density value within a row followed by the same letters are not significantly (p<0.05) different from each other. (DOCX) [file pone.0207762.s005.docx]

**S5 Table.** Shrub density (individual ha^-1^) across different Dzongri timberline sites of Khangchendzonga National Park

| Site | *Juniperus recurva* | *Rhododendron campanulatum* | *Rhododendron anthopogon* | *Rhododendron setosum* | *Rhododendron lepidotum* | *Ribes*  *glaciale* | *Rosa sericea* | *Gaultheria trichophylla* | *Gaultheria pyroloides* | Total |
| --- | --- | --- | --- | --- | --- | --- | --- | --- | --- | --- |
| Site 1 | 360±360 | 560±560 | 0.00±0.00 | 26.67±26.67 | 0.00±0.00 | 586.67±373.33 | 1207.11±108.48 | 0.00±0.00 | 0.00±0.00 | 2740±465^a^ |
| Site 2 | 400±400 | 0.00±0.00 | 440±440 | 960±960 | 0.00±0.00 | 232±232 | 353.33±73.33 | 0.00±0.00 | 0.00±0.00 | 2385±1495^ab^ |
| Site 3 | 0.00±0.00 | 26.67±26.67 | 0.00±0.00 | 142.22±142.22 | 186.67±186.67 | 136.0±68.04 | 826.67±254.38 | 0.00±0.00 | 0.00±0.00 | 1318±456^ab^ |
| Site 4 | 26.67±26.67 | 0.00±0.00 | 0.00±0.00 | 0.00±0.00 | 26.67±26.67 | 458.67±230.08 | 582.22±171.15 | 0.00±0.00 | 0.00±0.00 | 1094±431^ab^ |
| Site 5 | 240.0±138.56 | 0.00±0.00 | 0.00±0.00 | 80.0±80.0 | 0.00±0.00 | 115.56±35.56 | 408.89±155.75 | 0.00±0.00 | 0.00±0.00 | 844±240^ab^ |
| Site 6 | 0.00±0.00 | 553.33±286.67 | 0.00±0.00 | 80.0±80.0 | 0.00±0.00 | 180±20.0 | 540.0±0.00 | 0.00±0.00 | 0.00±0.00 | 1353±187^ab^ |
| Site 7 | 71.11±38.75 | 0.00±0.00 | 0.00±0.00 | 0.00±0.00 | 0.00±0.00 | 222.22±111.37 | 26.66±26.66 | 524.44±316.44 | 595.56±314.14 | 914±665^ab^ |
| Site 8 | 266.67±266.67 | 53.33.44±53.33 | 0.00±0.00 | 0.00±0.00 | 0.00±0.00 | 0.00±0.00 | 609.78±318.56 | 160.0±160.0 | 306.67±306.67 | 1303±396^ab^ |
| Site 9 | 117.33±23.25 | 86.67±6.67 | 0.00±0.00 | 0.00±0.00 | 0.00±0.00 | 160.0±87.18 | 93.33±48.07 | 53.33±53.33 | 0.00±0.00 | 511±72^b^ |
| Average | 164.64±51.89^b^ | 142.22±78.95^b^ | 48.89±48.89^b^ | 143.21±103.46^b^ | 23.70±20.58^b^ | 232.3560.29^ab^ | 516.44±120.41^a^ | 81.98±58.10^b^ | 100.25±70.54^b^ | 1365±205 |

Total shrub density values with in a column and average shrub density value within a row followed by same letters are not significantly (*p<0.05*) different from each other
