# Supplementary material for: Timberline structure and woody taxa regeneration towards treeline along latitudinal gradients in Khangchendzonga National Park, Eastern Himalaya
Source: PLoS One. 2018 Nov 28;13(11):e0207762. doi: 10.1371/journal.pone.0207762 (PMC6261585; doi:10.1371/journal.pone.0207762)
Supplement: S6 Table — Tree IVI values followed by the same letters with in a row are not significantly (p<0.05) different from each other among the sites. (DOCX) [file pone.0207762.s006.docx]

**S6 Table.** Important value index (IVI) of tree species across different Dzongri timberline sites of Khangchendzonga National Park

|  | *Abies densa* | *Sorbus microphylla* | *Rhododendron lanatum* | *Rhododendron wightii* | *Rhododendron hodgsonii* | *Rhododendron thomsonii* | *Rhododendron arboreum* | *Rhododendron fulgens* | *Piris*  *villosa* | *Prunus*  *rufa* |
| --- | --- | --- | --- | --- | --- | --- | --- | --- | --- | --- |
| Site 1 | 172.68±36.31^a^ | 51.54±13.73^bc^ | 65.95±37.27^b^ | 0.00±0.00^c^ | 0.00±0.00^c^ | 0.00±0.00^c^ | 0.00±0.00c | 0.00±0.00^c^ | 0.00±0.00^c^ | 9.83±5.58^c^ |
| Site 2 | 119.23±11.06^a^ | 59.40±9.79^b^ | 102.17±1.23^a^ | 8.35±8.35^c^ | 0.00±0.00^c^ | 0.00±0.00^c^ | 0.00±0.00^c^ | 0.00±0.00^c^ | 0.00±0.00^c^ | 10.85±10.85^c^ |
| Site 3 | 112.42±10.78^a^ | 102.07±11.32^a^ | 71.73±21.91^b^ | 0.00±0.00^c^ | 0.00±0.00^c^ | 0.00±0.00^c^ | 0.00±0.00^c^ | 0.00±0.00^c^ | 0.00±0.00^c^ | 13.79±13.79^c^ |
| Site 4 | 120.81±25.57^a^ | 39.00±34.39^bc^ | 81.27±23.44^ab^ | 39.84±13.55^bc^ | 0.00±0.00^c^ | 0.00±0.00^c^ | 0.00±0.00^c^ | 4.33±4.33^c^ | 0.00±0.00^c^ | 14.75±14.75^c^ |
| Site 5 | 68.29±36.07^ab^ | 87.01±25.09^a^ | 89.53±6.24^a^ | 34.84±13.55^bc^ | 0.00±0.00^c^ | 0.00±0.00^c^ | 0.00±0.00^c^ | 5.54±2.84^c^ | 4.10±4.10^c^ | 11.21±6.46^c^ |
| Site 6 | 111.12±8.23^a^ | 67.14±0.53^b^ | 45.12±11.98^cd^ | 0.00±0.00^e^ | 49.39±9.89^bc^ | 0.00±0.00^e^ | 0.00±0.00^e^ | 0.00±0.00^e^ | 0.00±0.00^e^ | 27.23±6.67^d^ |
| Site 7 | 45.14±37.91^a^ | 73.51±37.15^a^ | 61.36±13.94^a^ | 64.30±33.09^a^ | 0.00±0.00^a^ | 55.68±45.96^a^ | 0.00±0.00^a^ | 0.00±0.00^a^ | 0.00±0.00^a^ | 0.00±0.00^a^ |
| Site 8 | 50.28±16.09^abc^ | 79.52±32.22^a^ | 35.77±12.98^abcd^ | 27.73±9.93^bcd^ | 70.43±1.94^ab^ | 0.00±0.00^d^ | 13.60±6.80^cd^ | 0.00±0.00^d^ | 2.27±2.27^d^ | 20.40±16.50^cd^ |
| Site 9 | 0.00±0.00^c^ | 62.42±40.81^b^ | 14.52±14.52^bc^ | 155.71±10.05^a^ | 0.00±0.00^c^ | 0.00±0.00^c^ | 0.00±0.00^c^ | 0.00±0.00^c^ | 0.00±0.00^c^ | 67.34±37.61^b^ |
| Average | 88.88±17.38^a^ | 69.07±6.34^a^ | 63.85±8.67^a^ | 36.69±16.61^b^ | 13.31±8.98^bc^ | 6.19±6.19^c^ | 1.51±1.51^c^ | 1.09±0.73^c^ | 0.71±0.49^c^ | 19.49±6.48^bc^ |

Tree IVI values followed by same letters with in a row are not significantly (*p<0.05*) different from each other among the sites
