# Supplementary material for: Timberline structure and woody taxa regeneration towards treeline along latitudinal gradients in Khangchendzonga National Park, Eastern Himalaya
Source: PLoS One. 2018 Nov 28;13(11):e0207762. doi: 10.1371/journal.pone.0207762 (PMC6261585; doi:10.1371/journal.pone.0207762)
Supplement: S7 Table — Shrub IVI values followed by the same letters with in a row are not significantly (p<0.05) different from each other among the sites. (DOCX) [file pone.0207762.s007.docx]

**S7 Table.** Important value index (IVI) of shrub species across different Dzongri timberline sites of Khangchendzonga National Park

| Site | *Juniperus recurva* | *Rhododendron campanulatum* | *Rhododendron lepidotum* | *Rhododendron setosum* | *Rhododendron anthopogon* | *Ribes*  *glaciale* | *Rosa sericea* | *Gaultheria trichophylla* | *Gaultheria pyroloides* |
| --- | --- | --- | --- | --- | --- | --- | --- | --- | --- |
| Site 1 | 33.93±33.93^b^ | 39.45±39.45^b^ | 0.00±0.00 | 3.57±3.57^b^ | 0.00±0.00 | 50.68±36.59^b^ | 172.36±16.88^a^ | 0.00±0.00 | 0.00±0.00 |
| Site 2 | 26.17±26.17^ab^ | 0.00±0.00 | 35.99±35.99^ab^ | 59.78±59.78^ab^ | 0.00±0.00 | 80.89±80.89^a^ | 97.17±41.04^a^ | 0.00±0.00 | 0.00±0.00 |
| Site 3 | 0.00±0.00 | 8.364±8.36^ab^ | 0.00±0.00 | 18.20±18.20^ab^ | 28.63±28.63^ab^ | 61.96±34.38^b^ | 182.85±13.96^a^ | 0.00±0.00 | 0.00±0.00 |
| Site 4 | 8.93±8.93^b^ | 0.00±0.00 | 0.00±0.00 | 0.00±0.00 | 7.10±7.10^b^ | 56.31±35.15^b^ | 227.65±36.88^a^ | 0.00±0.00 | 0.00±0.00 |
| Site 5 | 63.91±32.82^b^ | 0.00±0.00 | 0.00±0.00 | 34.44±34.44^b^ | 0.00±0.00 | 65.559±23.88^b^ | 136.08±38.15^a^ | 0.00±0.00 | 0.00±0.00 |
| Site 6 | 0.00±0.00 | 143.66±21.24^a^ | 0.00±0.00 | 6.22±6.22^d^ | 0.00±0.00 | 49.97±6.13^c^ | 100.16±8.89^b^ | 0.00±0.00 | 0.00±0.00 |
| Site 7 | 40.93±22.75^bc^ | 0.00±0.00 | 0.00±0.00 | 0.00±0.00 | 9.57±9.57^bc^ | 36.41±27.22^bc^ | 118.06±09.48^a^ | 77.19±52.49^ab^ | 17.83±17.83^bc^ |
| Site 8 | 0.00±0.00 | 0.00±0.00 | 0.00±0.00 | 0.00±0.00 | 0.00±0.00 | 151.57±35.67^a^ | 114.99±29.69^a^ | 20.47±10.44^b^ | 12.97±12.97^b^ |
| Site 9 | 44.94±26.15^a^ | 56.843±29.65^a^ | 0.00±0.00 | 0.00±0.00 | 47.57±32.30^a^ | 29.69±13.25^a^ | 58.37±25.81^a^ | 5.81±5.81^a^ | 0.00±0.00 |
| Average | 24.31±7.80^c^ | 27.59±16.09^c^ | 3.998±3.99^c^ | 13.58±6.96^c^ | 10.32±5.61^c^ | 67.78±11.98^b^ | 134.19±17.27^a^ | 11.49±8.52^c^ | 3.42±2.30^c^ |

Shrub IVI values followed by same letters with in a row are not significantly (*p<0.05*) different from each other among the sites
