# Supplementary material for: Timberline structure and woody taxa regeneration towards treeline along latitudinal gradients in Khangchendzonga National Park, Eastern Himalaya
Source: PLoS One. 2018 Nov 28;13(11):e0207762. doi: 10.1371/journal.pone.0207762 (PMC6261585; doi:10.1371/journal.pone.0207762)
Supplement: S8 Table — (DOCX) [file pone.0207762.s008.docx]

**S8 Table. Relationship between environmental and ecological attributes of timberline across Khangchendzonga National Park, Eastern Himalaya**

|  | Elevation | Humus | Slope | TLD | Aspect | TDo | TDr | TR | TE | SDo | SDr | SR | SE | TD | SD | SAD | SHD | TBAT | TBAS |
| --- | --- | --- | --- | --- | --- | --- | --- | --- | --- | --- | --- | --- | --- | --- | --- | --- | --- | --- | --- |
| Elevation | 1 | -.739* | -0.149 | -0.528 | -0.258 | .823** | -.771* | -0.601 | -0.238 | -0.263 | 0.3 | 0.253 | -0.069 | -0.14 | -0.466 | -0.253 | -0.056 | -0.041 | -0.147 |
| Humus | -.739* | 1 | 0.412 | 0.597 | -0.313 | -.790* | .796* | 0.635 | 0.297 | -0.17 | 0.139 | 0.005 | -0.281 | -0.038 | 0.302 | -0.068 | -0.104 | -0.216 | -0.216 |
| Slope | -0.149 | 0.412 | 1 | 0.253 | -0.292 | -0.272 | 0.298 | 0.171 | -0.075 | -0.23 | 0.085 | -0.148 | 0.316 | 0.029 | 0.023 | 0.226 | 0.27 | 0.209 | 0.071 |
| TLD | -0.528 | 0.597 | 0.253 | 1 | -0.214 | -0.385 | 0.416 | 0.332 | 0.231 | -0.014 | -0.098 | -0.249 | -0.185 | -0.222 | 0.583 | 0.253 | 0.334 | -0.13 | 0.324 |
| Aspect | -0.258 | -0.313 | -0.292 | -0.214 | 1 | 0.176 | -0.304 | -0.371 | 0.071 | 0.455 | -0.383 | -0.052 | 0.227 | -0.147 | -0.309 | 0.026 | -0.065 | -0.042 | 0.159 |
| TDo | .823** | -.790* | -0.272 | -0.385 | 0.176 | 1 | -.979** | -.855** | -0.079 | -0.126 | 0.196 | 0.335 | -0.2 | -0.455 | -0.547 | -0.354 | -0.089 | -0.189 | 0.085 |
| TDr | -.771* | .796* | 0.298 | 0.416 | -0.304 | -.979** | 1 | .929** | -0.018 | 0.061 | -0.144 | -0.325 | 0.148 | 0.525 | 0.635 | 0.404 | 0.021 | 0.288 | -0.104 |
| TR | -0.601 | 0.635 | 0.171 | 0.332 | -0.371 | -.855** | .929** | 1 | -0.316 | 0.111 | -0.187 | -0.398 | 0.085 | .718* | .728* | 0.511 | -0.168 | 0.472 | -0.239 |
| TE | -0.238 | 0.297 | -0.075 | 0.231 | 0.071 | -0.079 | -0.018 | -0.316 | 1 | -0.479 | 0.511 | 0.614 | -0.12 | -.670* | -0.329 | -0.456 | 0.272 | -0.625 | 0.287 |
| SDo | -0.263 | -0.17 | -0.23 | -0.014 | 0.455 | -0.126 | 0.061 | 0.111 | -0.479 | 1 | -.974** | -.816** | 0.093 | 0.249 | 0.35 | 0.212 | 0.174 | 0.098 | 0.141 |
| SDr | 0.3 | 0.139 | 0.085 | -0.098 | -0.383 | 0.196 | -0.144 | -0.187 | 0.511 | -.974** | 1 | .909** | -0.238 | -0.328 | -0.478 | -0.39 | -0.316 | -0.234 | -0.258 |
| SR | 0.253 | 0.005 | -0.148 | -0.249 | -0.052 | 0.335 | -0.325 | -0.398 | 0.614 | -.816** | .909** | 1 | -0.329 | -0.488 | -0.642 | -0.581 | -0.4 | -0.378 | -0.188 |
| SE | -0.069 | -0.281 | 0.316 | -0.185 | 0.227 | -0.2 | 0.148 | 0.085 | -0.12 | 0.093 | -0.238 | -0.329 | 1 | 0.499 | 0.131 | .721* | 0.537 | 0.639 | 0.4 |
| TD | -0.14 | -0.038 | 0.029 | -0.222 | -0.147 | -0.455 | 0.525 | .718* | -.670* | 0.249 | -0.328 | -0.488 | 0.499 | 1 | 0.54 | .729* | -0.131 | .849** | -0.173 |
| SD | -0.466 | 0.302 | 0.023 | 0.583 | -0.309 | -0.547 | 0.635 | .728* | -0.329 | 0.35 | -0.478 | -0.642 | 0.131 | 0.54 | 1 | 0.664 | 0.282 | 0.502 | 0.351 |
| SAD | -0.253 | -0.068 | 0.226 | 0.253 | 0.026 | -0.354 | 0.404 | 0.511 | -0.456 | 0.212 | -0.39 | -0.581 | .721* | .729* | 0.664 | 1 | 0.318 | .856** | 0.317 |
| SHD | -0.056 | -0.104 | 0.27 | 0.334 | -0.065 | -0.089 | 0.021 | -0.168 | 0.272 | 0.174 | -0.316 | -0.4 | 0.537 | -0.131 | 0.282 | 0.318 | 1 | 0.014 | .775* |
| TBAT | -0.041 | -0.216 | 0.209 | -0.13 | -0.042 | -0.189 | 0.288 | 0.472 | -0.625 | 0.098 | -0.234 | -0.378 | 0.639 | .849** | 0.502 | .856** | 0.014 | 1 | 0.169 |
| TBAS | -0.147 | -0.216 | 0.071 | 0.324 | 0.159 | 0.085 | -0.104 | -0.239 | 0.287 | 0.141 | -0.258 | -0.188 | 0.4 | -0.173 | 0.351 | 0.317 | .775* | 0.169 | 1 |

* Correlation is significant at the 0.05 level

** Correlation is significant at the 0.01 level

TLD: treeline distance, TDo: tree dominance, TDr: tree diversity, TR: tree richness, TE: tree evenness, SDo: shrub dominance, SDr: shrub diversity, SR: shrub richness, SE: shrub evenness, TD: tree density; SD: seedling density, SAD: sapling density, SHD: shrub density, TBAT: total basal area tree, TBAS: total basal area shrub
