# Supplementary figures and images for: Timberline structure and woody taxa regeneration towards treeline along latitudinal gradients in Khangchendzonga National Park, Eastern Himalaya
Source: PLoS One. 2018 Nov 28;13(11):e0207762. doi: 10.1371/journal.pone.0207762 (PMC6261585; doi:10.1371/journal.pone.0207762)

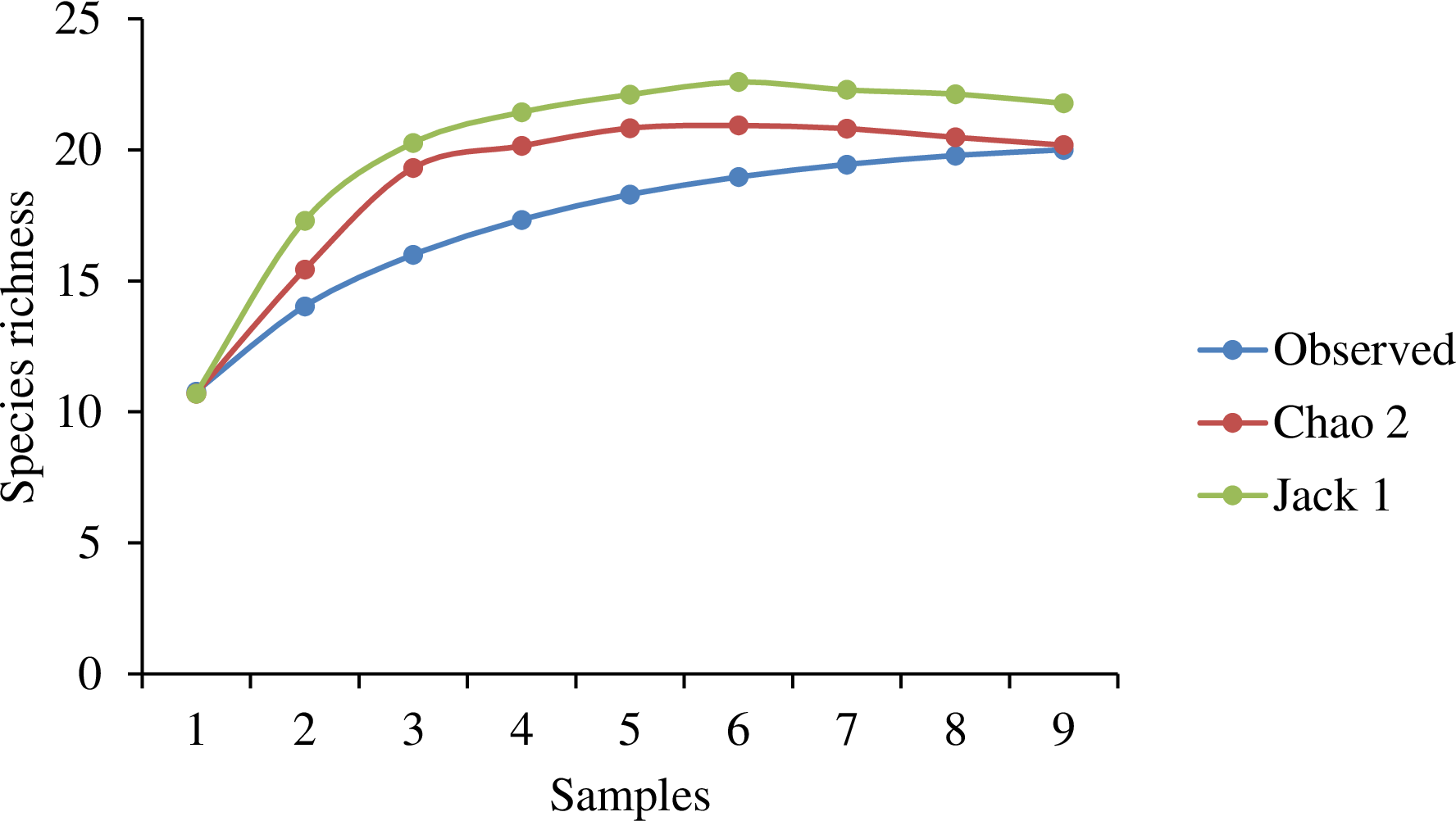

Supplement: S1 Fig — (TIF) [file pone.0207762.s009.tif]

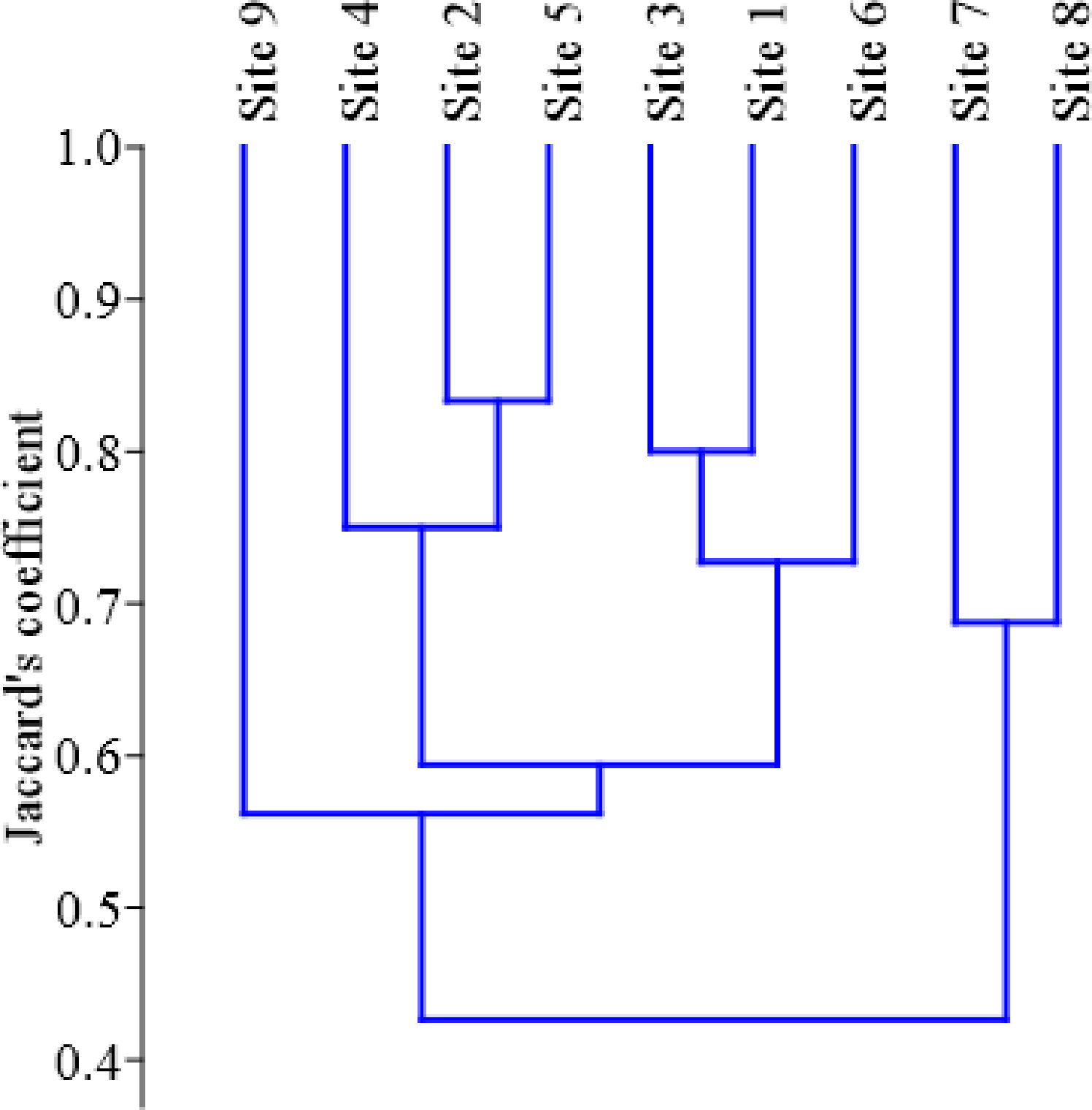

Supplement: S2 Fig — (TIF) [file pone.0207762.s010.tif]

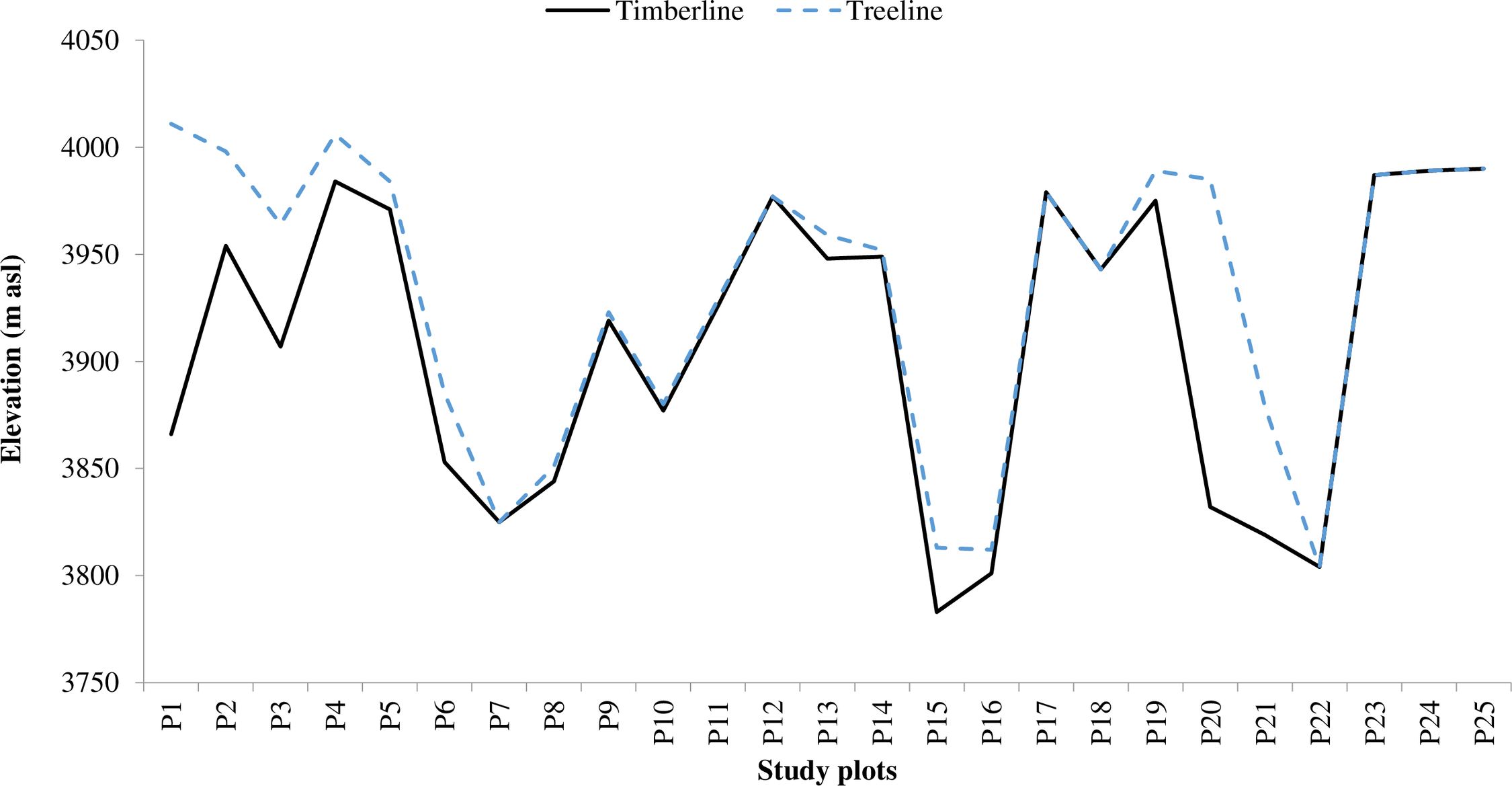

Supplement: S3 Fig — Plots (P1 to P25) were sampled (each of 50x20 m) across 9 sites (mostly 3 plots a site) over about 20 km. In timberline, tree crown cover is at least 30%, treeline connects the highest trees of each transect, distances between timberline and tree ranges from 0 to 155 m. At many sites treeline and timberline are the same, thus distance being 0. None of the seedlings and saplings extent beyond treeline, so treeline is unlikely to advance, however, the treeline and timberline ecotone can get densified. (TIF) [file pone.0207762.s011.tif]

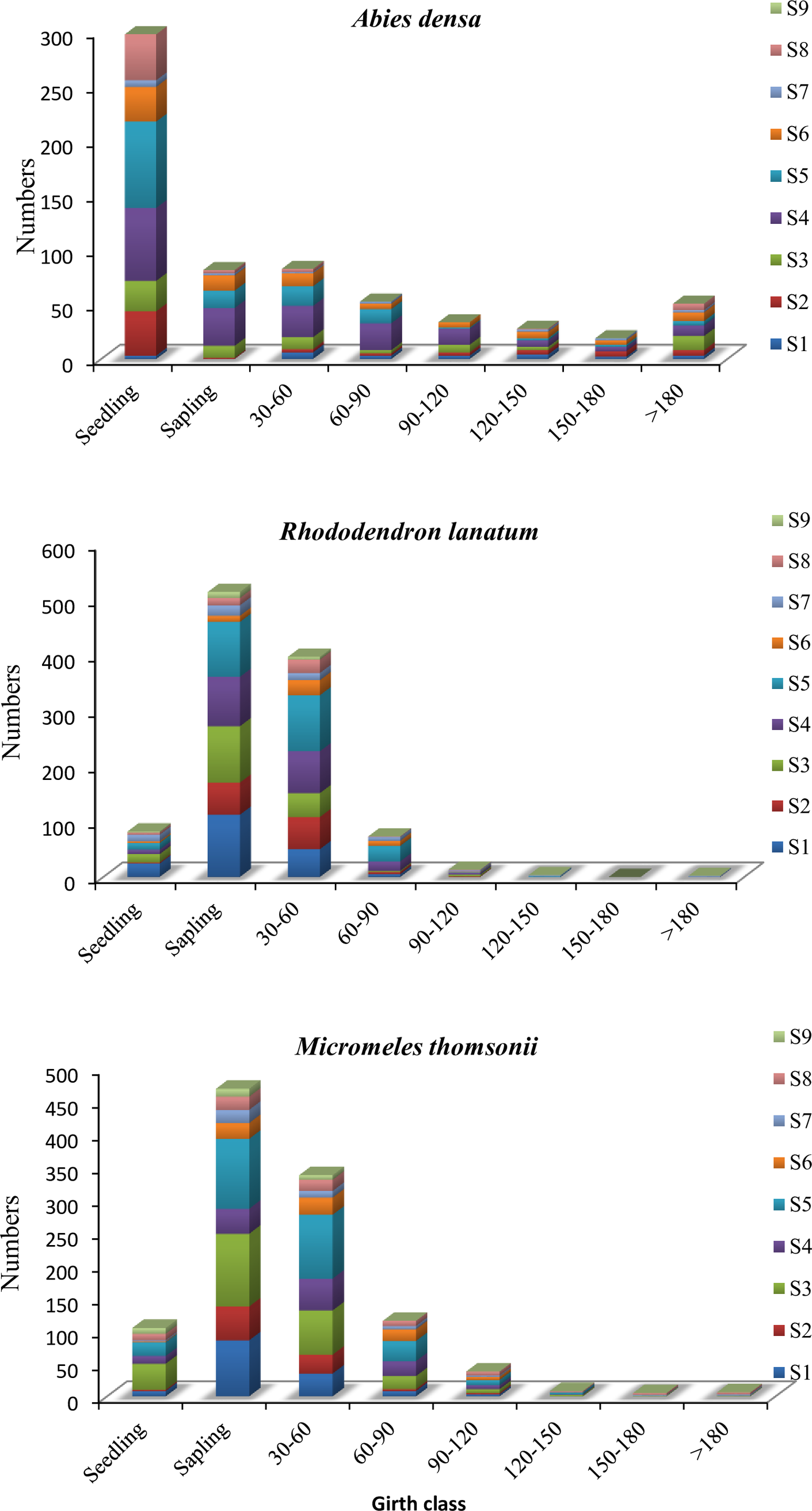

Supplement: S4 Fig — Rhododendron lanatum and Sorbus microphylla are showing good regeneration at studied sites, however Abies densa has shown poor conversion rate of seedlings in to the sapling or higher size class. Presence of individuals at higher girth class indicating historic presence of A. densa in timberline. (TIF) [file pone.0207762.s012.tif]

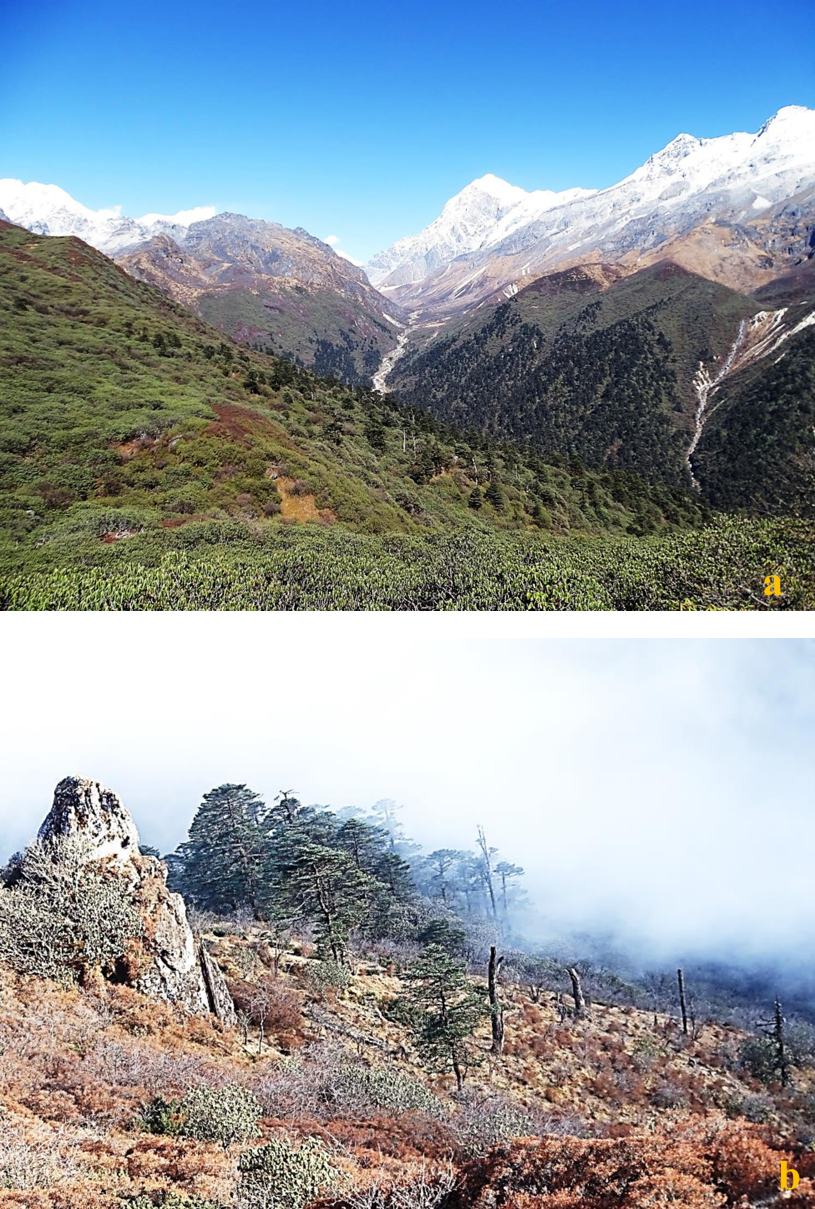

Supplement: S5 Fig — a. Orographic,; b. mechanical timberline boundaries. (TIF) [file pone.0207762.s013.tif]
